# Multiple Circulating Cytokines Are Coelevated in Chronic Obstructive Pulmonary Disease

**DOI:** 10.1155/2016/3604842

**Published:** 2016-07-25

**Authors:** Senthooran Selvarajah, Ian Todd, Patrick J. Tighe, Michelle John, Charlotte E. Bolton, Timothy Harrison, Lucy C. Fairclough

**Affiliations:** ^1^School of Life Sciences, University of Nottingham, Nottingham NG7 2UH, UK; ^2^Nottingham Respiratory Research Unit, School of Medicine, University of Nottingham, Nottingham NG5 1PB, UK

## Abstract

Inflammatory biomarkers, including cytokines, are associated with COPD, but the association of particular circulating cytokines with systemic pathology remains equivocal. To investigate this, we developed a protein microarray system to detect multiple cytokines in small volumes of serum. Fourteen cytokines were measured in serum from never-smokers, ex-smokers, current smokers, and COPD patients (GOLD stages 1–3). Certain individual circulating cytokines (particularly TNF*α* and IL-1*β*) were significantly elevated in concentration in the serum of particular COPD patients (and some current/ex-smokers without COPD) and may serve as markers of particularly significant systemic inflammation. However, numerous circulating cytokines were raised such that their combined, but not individual, elevation was significantly associated with severity of disease, and these may be further indicators of, and contributors to, the systemic inflammatory manifestations of COPD. The coelevation of numerous circulating cytokines in COPD is consistent with the insidious development, chronic nature, and systemic comorbidities of the disease.

## 1. Introduction

Chronic obstructive pulmonary disease (COPD) is a major cause of morbidity and mortality worldwide. It is induced mainly by tobacco smoking, which leads to chronic, irreversible lung inflammation; both cellular and humoral immunological processes are involved in the pathology [1–6]. A significant proportion of patients also have other disease manifestations (e.g., cardiovascular disease, metabolic disease, bone disease, and cachexia), which are associated with systemic inflammation [1, 7–10]. Inflammatory biomarkers, including cytokines, are associated with both the lung disease and the systemic pathology [1, 11–22]. These circulating cytokines could be “spill over” from lung inflammation or induced separately from pathological processes in the lung; evidence for the latter is differences in cytokine profiles seen in the lung and circulation, and only a proportion of COPD patients show a significant rise in individual circulating cytokines [1].

Cytokines are not disease-specific biomarkers, as they may be raised for many different reasons (e.g., infection, autoimmunity, and intrinsic inflammation), which may be subclinical; therefore, some never-smokers with no overt clinical disease may have raised cytokines, as well as smokers and COPD patients. This is one of the reasons why it is difficult to identify particular individual circulating cytokines as disease-specific markers for COPD.

Some previous studies have investigated a broad range of systemic cytokines in COPD [11–14], but others have examined only a few. In general, relatively large cohorts of patients and controls have been required to demonstrate statistically significant elevation of individual cytokines in COPD, and different studies have concluded that different cytokines are raised [15–20]. This suggests that systemic inflammation in COPD occurs in only a proportion of patients with lung disease (as evidenced by others, e.g., [15]) and/or a subtle, broad elevation of numerous cytokines is associated with the systemic inflammation. To investigate this further, we developed an in-house protein microarray system to detect multiple cytokines in small volumes of serum. The system was rigorously validated for accuracy and reproducibility and reported previously in this journal [23]. Patients with mild, moderate, or severe COPD [GOLD (global initiative for chronic obstructive lung disease) stages 1–3] were investigated in comparison with never-smokers (NS) and current smokers without COPD (CS).

## 2. Materials and Methods

### 2.1. Subjects

Serum was obtained from 57 patients with confirmed COPD, 25 ex-smokers without COPD (ExS), 9 current smokers without COPD (CS), and 11 never-smoker (NS) healthy controls. Subjects were recruited from volunteer databases and respiratory out-patient clinics and by advertisement. All subjects were over 40 years of age, of European descent, had a significant smoking history of greater than 10 pack years, and were studied at clinical stability (no change in regular therapy, no requirement for antibiotics and/or oral corticosteroids in the preceding 4 weeks, and no change in symptoms beyond day-to-day variation). All subjects gave written informed consent and the study was approved by the National Research Ethics Committee [Leicestershire, Northamptonshire, Rutland (LNR), REC 10/H0406/65]. No subject recruited had active or suspected malignancy, terminal disease, or known alpha-1-antitrypsin deficiency. Samples were collected and stored at −70°C until used. The demographics of the groups are shown in Table 1 and described in Section 3.

### 2.2. Cytokine Microarray

**Figure 1:**
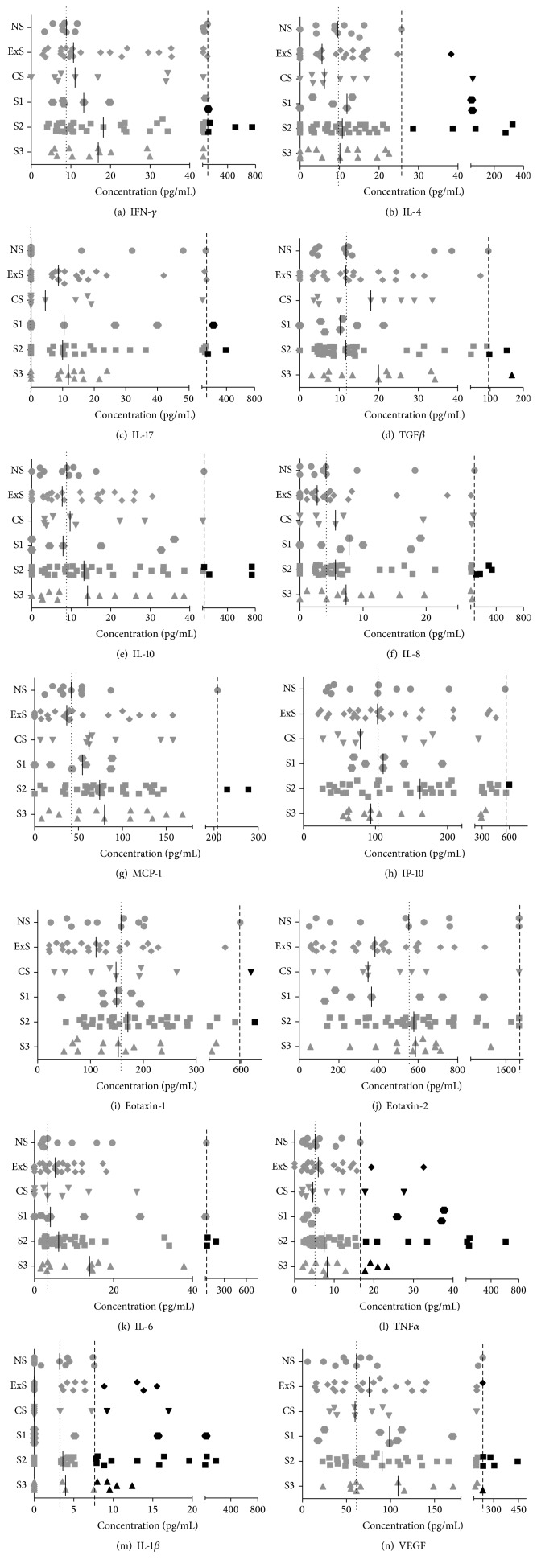
Concentrations of 14 cytokines in serum samples from control subjects and patients with COPD. The number of subjects in each group was never-smokers (NS), 11; ex-smokers (ExS), 25; current smokers (CS), 9; COPD GOLD stage 1 (S1), 7; COPD S2, 37; COPD S3, 13. Cytokines were measured by protein microarray (see text and [23] for details). Experiments were performed three times, with two replicates in each experiment, and the data averaged across the three experiments. The data are presented as scatter plots, with each point representing an individual subject's serum sample, and the solid, short vertical lines show the median values for each cytokine in each group. The dotted vertical lines represent the median values of the NS group. The dashed vertical lines represent the 95th percentile of the NS group: individual cytokine concentrations higher than this are shown by black symbols and those below by grey symbols.

Serum cytokine concentrations were determined by protein microarray, as described in detail previously in this journal [23]. Briefly, capture antibodies from DuoSet kits (R&D Systems, MN, USA) were printed onto poly-L-lysine slides. The slides were blocked with either I-Block or 3% BSA block buffer. Standards were made for all 14 cytokines (see Figure 1) according to the manufacturer's instructions and 7 twofold dilutions were applied to the microarrays. Additionally, the NS, CS, and COPD sera were added to microarray slides. The slides were washed and 50 *μ*L of appropriately diluted DuoSet biotinylated detection antibody (R&D Systems, MN, USA) were added, followed by streptavidin-HRP (Bio-Rad, USA). The slides were washed and 50 *μ*L of Bio-Rad Amplification Reagent (Opti-4CN amplification kit) was added for 10 minutes in the dark. The slides were washed extensively with 20% DMSO-PBST and with wash buffer, followed by the addition of streptavidin-conjugated cy5 (E-Biosciences, UK). The slides were scanned with a 4200 AL microarray scanner at 635 nm (Axon GenePix®). Fluorescence was quantified using the GenePix Pro Software (Axon GenePix). The median fluorescence of each spot was measured (minus background) and the corrected fluorescence was used to calculate the average fluorescence signal across the standard detectable ranges.

### 2.3. Statistical Analyses

The data were not normally distributed and therefore multiple comparisons between groups were made using the Kruskal-Wallis test for nonpaired data (5 comparisons) and the Friedman test for paired data (13 or 14 comparisons); in both cases Dunn's post hoc test for multiple comparisons was applied. Statistical analyses were performed using GraphPad Prism 6 (La Jolla, California).

## 3. Results

### 3.1. Demographics of Study Groups

The demographics of the subjects who donated serum for this study are shown in Table 1. There were no significant age differences between groups except for the never-smokers (NS) compared to the COPD GOLD stage 2 (S2) patients, with the NS subjects being slightly younger. Furthermore, serum levels of most cytokines do not differ significantly between younger and older adults [24]. Although the gender ratio varied, there are no significant differences in peripheral blood cytokine concentrations between females and males [25]. Patients with moderate or severe COPD (GOLD stages 2-3) had significantly lower FEV1-% than never-smokers or current/ex-smokers without COPD.

### 3.2. Effects of Smoking Status on Cytokine Levels

Fourteen cytokines were measured in serum from never-smokers (NS, 11 subjects), ex-smokers without COPD (ExS, 25 subjects), current smokers without COPD (CS, 9 subjects), and COPD patients at GOLD stages 1–3 (S1, 7 subjects; S2, 37 subjects; S3, 13 subjects) by protein microarray (Figure 1). [One NS subject and one CS subject were excluded from the above groups because they had extremely elevated levels of numerous cytokines compared to all other members of the group (i.e., concentrations more than ×3 higher than any other subjects in the groups). It was therefore judged that their exceptionally high cytokine levels may have been indicative of other undiagnosed pathological processes, so they could not be considered representative of the NS and CS groups.]

**Figure 2:**
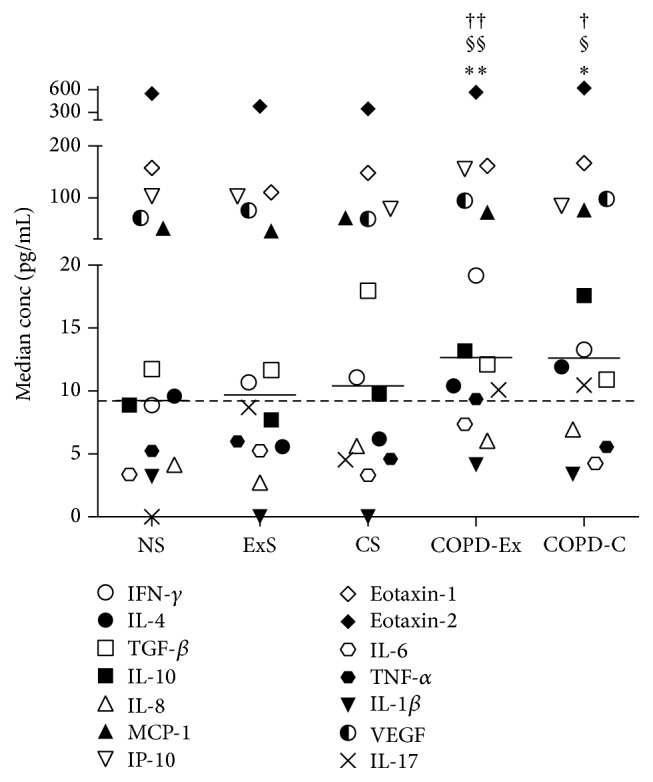
Median concentrations of 14 cytokines in serum samples from the following groups of subjects: NS (*n* = 11), ExS (*n* = 25), CS (*n* = 9), COPD ex-smokers (COPD-Ex, *n* = 42), and COPD current smokers (COPD-C, *n* = 15). The symbol representing each cytokine is shown in the figure. The solid, short horizontal lines represent the median concentrations of the 14 cytokines in each group. The dashed horizontal line represents the median value of the NS group. COPD-Ex and COPD-C were significantly different from NS (*∗*), ExS (§), and CS (†). There were no significant differences between NS, ExS, and CS or between COPD-Ex and COPD-C. *∗* refers to significant differences between the NS group and the COPD-Ex or COPD-C groups. § refers to significant differences between the ExS group and the COPD-Ex or COPD-C groups. † refers to significant differences between the CS group and the COPD-Ex or COPD-C groups. The number of symbols refer to the level of statistical significance, so one symbol (*∗*, §, †) indicates *p* = 0.03; two symbols (*∗∗*, §§, ††) indicate *p* = 0.002; three symbols (*∗∗∗*, §§§, †††) indicate *p* = 0.0002; four symbols (*∗∗∗∗*, §§§§, ††††) indicate *p* < 0.0001; these values were determined by Friedman test followed by Dunn's multiple comparisons test. The same applies to Figures 3 and 4.

The majority of the COPD patients at all GOLD stages were ex-smokers (COPD-Ex) and a minority were current smokers (COPD-C). However, as shown in Figure 2, there were no significant differences in the median levels of the 14 cytokines between the COPD-Ex and COPD-C groups, although both of these groups had significantly higher levels of cytokines overall compared to the NS (*∗*), ExS (§), and CS (†) groups (Friedman test with Dunn's post hoc test) (Figure 2). This indicates that the elevation of serum cytokine concentrations in the patients was principally a consequence of COPD rather than their smoking status. Further comparisons were therefore made between the NS, CS, and S1–3 groups in order to identify the effects of smoking* per se* (in the CS subjects), as well as the effects of the severity of COPD (in the S1–S3 subjects), on serum levels of the 14 cytokines studied.

### 3.3. Significant Elevation of Particular Serum Cytokines in Individual Subjects

Figure 1 shows that numerous cytokines were elevated in CS and, increasingly so, in S1 to S3; however, none of these changes were statistically significant for particular cytokines in a group of subjects as a whole compared to the NS group (Kruskal-Wallis test with Dunn's post hoc test). This could be partly because the levels of most cytokines vary greatly, even in NS subjects, as is apparent in Figure 1. However, it could also be because only a proportion of patients have a statistically significant rise of particular cytokines, as has been reported by others [15], and suspected to correspond with those patients with systemic inflammatory disease. Therefore, as in previous studies, we defined individual patients as showing significant elevation of particular cytokines if their serum concentration exceeded the 95th percentile of the NS controls [15]. This is shown for each cytokine in Figure 1, where the dashed lines represent 95th percentile of the serum cytokine concentrations in the NS group; subjects with cytokine concentrations higher than this are shown by black symbols, and those with lower concentrations are shown by grey symbols. Overall, at least one cytokine was significantly elevated (i.e., >95th percentile of NS) in 22% CS, 43% S1, 43% S2, and 54% S3 subjects, 28 out of 66 subjects in total, that is, 42% positive. Fifteen of the 28 positives had 2 or more cytokines raised, and several had multiple cytokines raised, particularly in S2 subjects (the highest being 11/14 cytokines raised in one subject). The cytokines that were most frequently significantly elevated were TNF*α* (Figure 1(l)) and IL-1*β* (Figure 1(m)); together, these detected 23/28 positives; this is partly because the variations in levels of TNF*α* and IL-1*β* in NS subjects were relatively small, thereby generating low cut-off values for the 95th percentile (Figures 1(l) and 1(m), resp.). Out of the other five positives, two had elevated IL-8 (Figure 1(f)), two VEGF (Figure 1(n)), and one TGF*β* (Figure 1(d)). Several subjects also had elevated levels of IL-4 (Figure 1(b)), but all of these also had elevated TNF*α* and IL-1*β*.

### 3.4. Significant Summative Elevation of Multiple Serum Cytokines in Subject Groups

**Figure 3:**
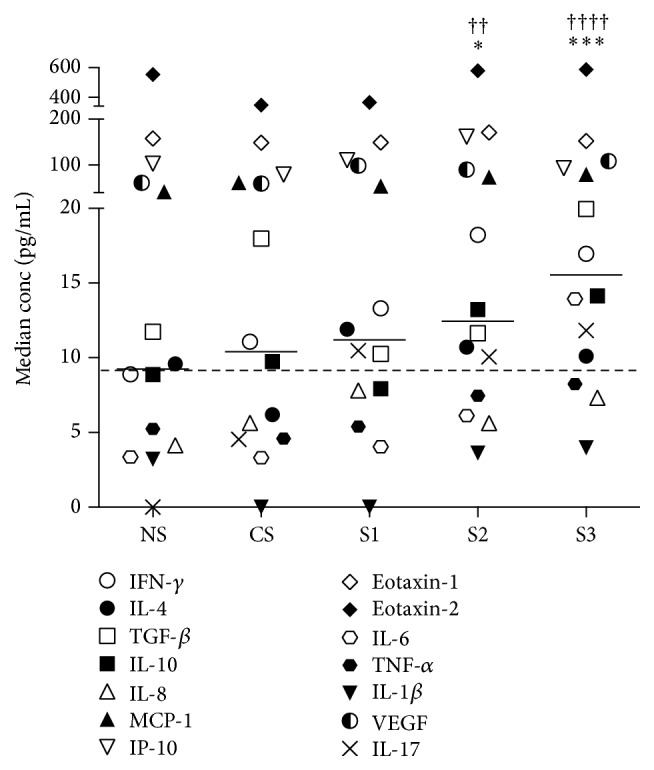
Median concentrations of 14 cytokines in serum samples from the following groups of subjects: NS (*n* = 11), CS (*n* = 9), COPD S1 (*n* = 7), COPD S2 (*n* = 37), and COPD S3 (*n* = 13). Other details are as described in the legend to Figure 2. Compared to the NS group, cytokines concentrations were significantly higher in the S2 (*∗*) and S3 (*∗∗∗*) groups; similarly, compared to the CS group, cytokines concentrations were significantly higher in the S2 (††) and S3 (††††) groups.

The solid bars on the graphs in Figure 1 represent the median serum concentrations of the cytokines in each group of subjects.  Figure 3 shows a plot of these median cytokine concentrations for all 14 cytokines in the NS, CS, and S1–3 groups. It can be seen that, compared to NS, there is a gradual, general increase of serum cytokine concentrations with increasing disease severity (CS < S1 < S2 < S3). Furthermore, the collective serum cytokine concentrations in S2 and S3 were significantly raised compared to both NS (*∗*) and CS (†) (Friedman test with Dunn's post hoc test).

This generalised increase in serum cytokines was further investigated by identifying subjects with serum cytokine concentrations higher than the 50th percentile (i.e., median) concentrations seen in the NS control group, which are shown for each cytokine by the dotted lines in the graphs in Figure 1. By definition, less than 50% of the NS subjects had concentrations above the NS group median values for all cytokines. By contrast, Figure 1 shows that the median levels of several cytokines (shown by the solid bars) are higher than the NS median in the CS and COPD groups. Indeed, in the CS group and COPD S1, S2, and S3 groups, several cytokines had serum concentrations greater than the NS group median values in at least two-thirds (≥67%) of the subjects: this was the case for MCP-1 in all four groups (CS and S1–3); for IL-8 in the S1–3; for VEGF in S1 and S2; for IFN-*γ*, IL-6 and TNF*α* in S2 and S3; and for IL-17 in S3 only.

**Figure 4:**
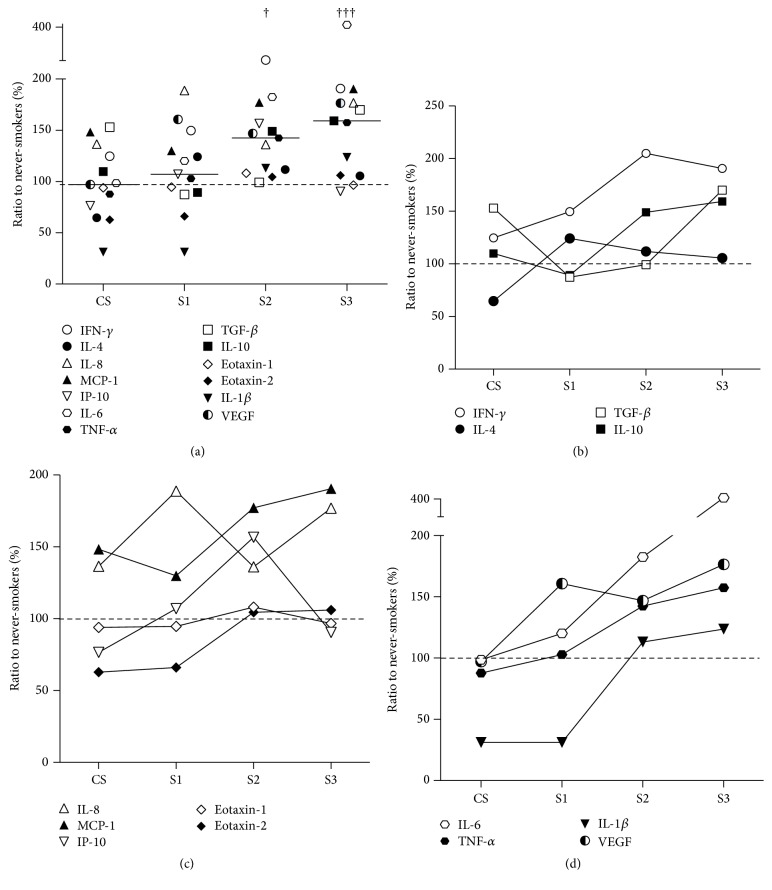
(a) Ratios of the median concentrations of each serum cytokine in the CS, S1, S2 and S3 groups expressed as percentages of these median concentrations in the NS control group. The symbol representing each cytokine is as shown in (b)–(d). The solid, short horizontal lines represent the median ratios to NS of the 13 cytokines in each group. The dashed horizontal line, which represents the median ratio to NS of the CS group. Compared to the CS group, cytokine ratios to NS were significantly higher in the S2 (†) and S3 (†††) groups. (b)–(d) Representations of the data in (a) presented to facilitate analysis of the relative difference in expression of each serum cytokine between the CS, S1, S2 and S3 groups, divided into “Th1/Th2/Treg” cytokines (b), “chemokines” (c) and “inflammatory cytokines and growth factor” (d). The dashed horizontal lines represent the standardised cytokine concentrations in the NS group (i.e., 100%).

Figure 4(a) presents the ratios of the median concentrations of each cytokine in each group relative to the NS control group. Collectively, these values are significantly higher in the S2 (†) and S3 (†††) groups compared to the CS group (Friedman test with Dunn's post hoc test). Figures 4(b)–4(d) show plots of these ratios for each cytokine separated into “Th1/Th2/Treg” cytokines (Figure 4(b)), “chemokines” (Figure 4(c)) and “inflammatory cytokines and growth factor” (Figure 4(d)). The symbol for each cytokine is indicated in the figure; IL-17 (× in Figures 2 and 3) is not shown in Figures 4(b)–4(d) because the median of the NS group was 0 (Figure 1(c)), so ratios could not be derived.

## 4. Discussion

Our findings are consistent with those of others who have shown elevated levels of serum cytokines in COPD, particularly with regard to IL-6, IL-8, TNF*α*, and VEGF [11–16, 18, 20]. We found certain individual circulating cytokines (particularly TNF*α* and IL-1*β*) to be significantly elevated in concentration in the serum of some COPD patients (and some current smokers not diagnosed with COPD), and these may serve as markers of particularly significant systemic inflammation. However, we also found a broad increase in the serum concentration of numerous circulating cytokines that was collectively significant (and of possible biological relevance), but without a sufficiently great increase in concentration across all patients being statistically significant for individual cytokines. Hence, this indicates the value of detecting numerous cytokines rather than just a few. The serum levels of this collection of cytokines correlated with the severity of COPD (GOLD stages 1–3), and they may be further indicators of, and contributors to, the systemic inflammatory manifestations of COPD.

The broad, relatively subtle, elevation of multiple circulating cytokines is also consistent with the insidious development and chronic nature of COPD. Indeed, this pattern of moderate, but persistent cytokine elevation may also occur in other chronic conditions, including metabolic syndrome and cardiovascular disease, which are also associated with COPD [26, 27].

A more detailed analysis of the trends in cytokine expression represented in Figures 4(b)–4(d) raises a number of interesting points. Firstly, some cytokines appear to be elevated as a consequence of smoking* per se*, particularly, IFN-*γ*, TGF*β*, IL-8, and MCP-1. Secondly, most of the cytokines studied are elevated in COPD patients, particularly in S2 and S3; this applies to all except IL-4, eotaxin-1, and eotaxin-2 (i.e., Th2-profile cytokines). Thirdly, only VEGF is elevated in all three stages of COPD (S1–3) relative to CS. This is consistent with other reports of the particular importance of VEGF in COPD and the fact that hypoxia is a known inducer of VEGF [28–30]. Proinflammatory cytokines, including ones found to be elevated in the present study, are known inducers of VEGF expression by alveolar epithelial cells [31]. Finally, IL-1*β* appears to be suppressed in CS and S1. At first site, this appears surprising given that IL-1*β* (together with TNF*α*) was the best discriminator of patients with cytokine levels >95th percentile of the NS group. However, this is because CS and COPD subjects tend to have either low levels of IL-1*β* or high levels (above 95th percentile of NS), rather than an even spread (as shown in Figure 1(m)). Indeed, it is interesting to note that the serum cytokines IL-1*β* and IL-4, which were significantly elevated (above the 95th percentile of the NS controls) in certain individual subjects, were not generally raised in the CS or COPD groups. This might be because these cytokines are associated with particular disease manifestations rather than the generalised, systemic, low-grade inflammation associated with COPD. Our study suggests that the latter phenotype is more associated with IFN-*γ*, IL-17, MCP-1, IL-8, VEGF, IL-6, and TNF*α* from amongst the cytokines we measured.

The findings of the present study can be put into the context of “systems biology” of disease processes, in which a combination of technological and analytical advances has facilitated understanding of the complexity of factors that contribute to pathogenesis and phenotype of diseases. This, in turn, has led to the realisation that multiple, interacting factors can have a significant impact on disease development in combination, but not individually. Examples in other types of disease include the subtle, constitutive activation of multiple proinflammatory signalling pathways in certain autoinflammatory disorders [32] and patterns of reactivity of multiple natural autoantibodies with tumours [33].

## 5. Conclusions

In conclusion, numerous cytokines are raised in the circulation of COPD patients such that the combined, but not individual, elevation of these cytokines is significantly associated with severity of disease. Furthermore, these cytokines may be indicators of, and contributors to, the systemic inflammatory manifestations of COPD and the pattern of multiple serum cytokines may be more informative of systemic aspects of the disease than individual cytokines. The coelevation of numerous circulating cytokines in COPD is consistent with the insidious development, chronic nature, and systemic comorbidities of this disease.

## Figures and Tables

**Table 1:** Demographic data of subjects.

Group	Never-smokers (NS)	Ex-smokers (ExS)	Current smokers (CS)	COPD GOLD stage 1 (S1)	COPD GOLD stage 2 (S2)	COPD GOLD stage 3 (S3)
Number	11	25	9	7	37	13
Age (yrs)						
Median (range)^*∗*^	56 (41–67)	71 (42–80)	69 (42–76)	65 (61–82)	68 (46–82)	67 (47–84)
F/M	6/5	12/13	3/6	2/5	18/19	5/8
FEV1-%						
Median (range)^§^	97 (87–134)	98 (74–124)	96 (84–115)	87 (80–103)	62 (51–77)	42 (30–49)
Ex-smokers	0	25	0	4	30	8
Current smokers	0	0	9	3	7	5

^*∗*^The age of the NS group was significantly different from S2; there were no other significant age differences between groups (by Kruskal-Wallis test with Dunn's post hoc test for multiple comparisons).

^§^The NS, ExS, and CS groups were all significantly different from both S2 and S3 for FEV1-% of predicted, and S1 was significantly different from S3; there were no other significant differences between groups (by Kruskal-Wallis test with Dunn's post hoc test for multiple comparisons).
